# Subjective and objective health predicting mortality and institutionalization: an 18-year population-based follow-up study among community-dwelling Finnish older adults

**DOI:** 10.1186/s12877-021-02311-w

**Published:** 2021-06-10

**Authors:** Anna Viljanen, Marika Salminen, Kerttu Irjala, Elisa Heikkilä, Raimo Isoaho, Sirkka-Liisa Kivelä, Päivi Korhonen, Tero Vahlberg, Matti Viitanen, Maarit Wuorela, Minna Löppönen, Laura Viikari

**Affiliations:** 1 Municipality of Lieto, Health Care Center, Hyvättyläntie 7, 21420 Lieto, Finland; 2 Faculty of Medicine, Department of Clinical Medicine, Unit of Geriatrics, FI-20014 University of Turku, Turku City Hospital, Kunnallissairaalantie 20, 20700 Turku, Finland; 3 City of Turku, Welfare Division, Yliopistonkatu 30, 20101 Turku, Finland; 4 Faculty of Medicine, Department of Clinical Medicine, Unit of Family Medicine, University of Turku and Turku University Hospital, 20014 Turku, Finland; 5 Faculty of Medicine, Department of Clinical Medicine, Unit of Clinical Chemistry, TYKSLAB, 20521 Turku, Finland; 6 City of Vaasa, Social and Health Care, Ruutikellarintie 4, 65101 Vaasa, Finland; 7 Faculty of Pharmacy, Division of Social Pharmacy, University of Helsinki, 00014 Helsinki, Finland; 8 Faculty of Medicine, Department of Clinical Medicine, Unit of Biostatistics, University of Turku, Turku, Finland; 9 Division of Clinical Geriatrics, Center for Alzheimer Research, Department of Neurobiology, Care Sciences and Society, Karolinska Institutet and Karolinska University Hospital, Huddinge, Stockholm, Sweden; 10 City of Raisio, Social and Health Care for Elderly, Sairaalakatu 5, 21200 Raisio, Finland

**Keywords:** Institutionalization, Mortality, Objective health, Older people, Registered illnesses, Self-rated health, Subjective health

## Abstract

**Background:**

Objective health measures, such as registered illnesses or frailty, predict mortality and institutionalization in older adults. Also, self-reported assessment of health by simple self-rated health (SRH) has been shown to predict mortality and institutionalization. The aim of this study was to assess the association of objective and subjective health with mortality and institutionalization in Finnish community-dwelling older adults.

**Methods:**

In this prospective study with 10- and 18-year follow-ups, objective health was measured by registered illnesses and subjective health was evaluated by simple SRH, self-reported walking ability (400 m) and self-reported satisfaction in life. The participants were categorized into four groups according to their objective and subjective health: 1. subjectively and objectively healthy, 2. subjectively healthy and objectively unhealthy, 3. subjectively unhealthy and objectively healthy and 4. subjectively and objectively unhealthy. Cox regression model was used in the analyses. Death was used as a competing factor in the institutionalization analyses.

**Results:**

The mean age of the participants (*n* = 1259) was 73.5 years (range 64.0–100.0). During the 10- and 18-year follow-ups, 466 (37%) and 877 (70%) died, respectively. In the institutionalization analyses (*n* = 1106), 162 (15%) and 328 (30%) participants were institutionalized during the 10- and 18-year follow-ups, respectively. In both follow-ups, being subjectively and objectively unhealthy, compared to being subjectively and objectively healthy, was significantly associated with a higher risk of institutionalization in unadjusted models and with death both in unadjusted and adjusted models.

**Conclusions:**

The categorization of objective and subjective health into four health groups was good at predicting the risk of death during 10- and 18-year follow-ups, and seemed to also predict the risk of institutionalization in the unadjusted models during both follow-ups. Poor subjective health had an additive effect on poor objective health in predicting mortality and could therefore be used as part of an older individual’s health evaluation when screening for future adverse outcomes.

**Supplementary Information:**

The online version contains supplementary material available at 10.1186/s12877-021-02311-w.

## Background

Self-rated health (SRH) is an individual’s own perception of their health. It is a subjective assessment, but still has been shown to predict mortality in older adults [1, 2]. The association has been shown in different age groups [3], even in the very old (> 90 years) population [4], and in different ethnicities [5, 6]. There are, however, differences between cultures as to how individuals perceive their health [7]. The predictive ability of SRH on mortality has been identified in both genders [3, 8], although differences have been found between genders [9–11].

The association of multimorbidity and mortality is commonly assessed using multimorbidity indices. A systematic review [12] suggests that the Charlson Comorbidity Index (CCI) [13] has the strongest evidence for studying the relationship between multimorbidity and mortality. However, disease counts have in some studies been found almost as effective at predicting mortality [12], and also a new simpler comorbidity index for use in the primary care setting has been suggested on the basis of disease count [14]. In both the CCI and the simpler index, the illnesses have been assigned a weight according to severity. However, of the illnesses chosen, each alone predicted a higher risk of mortality suggesting that even one moderate to severe illness could predict an increased risk of mortality [13, 14].

The association of poor SRH and institutionalization has been shown in earlier studies [15, 16]. Also poor objective health at 70-years, assessed by the number of illnesses and medications used, has been shown to predict institutionalization [17]. An earlier study compared the concordance of SRH and physician rated health (PRH) (based on registered illnesses), and their ability to predict institutionalization, and found SRH a better predictor of institutionalization [18].

We have earlier analyzed the association of frailty with mortality [19], and simple SRH, the self-reported ability to walk 400 m and frailty with institutionalization, and found that those two simple self-reported items predicted institutionalization almost as well as frailty during a 10-year follow-up period [16]. An earlier study found that having the combination of poor SRH and poor PRH was associated with a higher risk of death than the combinations of having poor SRH but good PRH or having good SRH but poor PRH [20]. The aim of the current study was to analyze the association of a broader self-evaluation of health (including SRH, satisfaction in life and the self-reported ability to walk 400 m) and an objective measure of health (registered illnesses) with mortality and institutionalization in a population of community-dwelling Finnish older adults. Our main interest was to analyze the risk of adverse effects associated with different combinations of subjective and objective health, and their interaction.

## Methods

### Study design and population

This study is a part of the longitudinal epidemiological study carried out in the municipality of Lieto in southwest Finland [21]**.** All persons born in or prior to the year 1933 (*n* = 1596) were invited to participate in the baseline examination that took place between March 1998 and September 1999. Of those eligible, 63 died before they were examined and 273 refused or did not respond leaving 1260 (82%) participants, 533 men and 727 women.

At baseline the study protocol consisted of an extensive interview on demographic and socioeconomic factors and health behavior, numerous laboratory tests, and a clinical examination including a comprehensive survey of the participants’ medical records [21].

An outlier, institutionalized in year 1930 at the age of 17, was excluded from the analyses leaving 1259 participants for the mortality analyses. Participants no longer living in Lieto at the end of 2016 (*n* = 86) were excluded from the analyses on institutionalization, as it was not possible to ascertain whether they continued living at home or were institutionalized in another municipality. Also, participants already living in institutional care (*n* = 67) at baseline were excluded. Exclusions left us with 1106 participants for the institutionalization analyses.

### Subjective health

To be classified as comprehensively healthy on self-report data (*n* = 420), the participant had to meet all the following criteria: self-rated health very good or good, self-reported satisfaction in life very good or good, and self-reportedly able to walk 400 m independently, with or without difficulties, the data of which were gathered at baseline by the questions: “How would you rate your current state of health?” with the answer options of “very good”, “good”, “moderate”, “poor” and “very poor”, “How would you rate your current satisfaction in life?” with the answer options of “very good”, “good”, “moderate”, “poor” and “very poor” and “Are you able to walk at least 400 meters?” with the answer options of “yes, without difficulty”, “yes, with difficulty”, “with help” and “no”.

### Objective health

We used the baseline data of the participants’ registered illnesses, gathered from patient records and clinical examination, to evaluate their general health. The illnesses were classified according to ICD-10 (Additional file 1) and participants were classified as objectively healthy if they didn’t have any of the illnesses (*n* = 310). The cut-off was chosen to best identify the subjectively and objectively healthy, the “super healthy” participants.

### Combined health information

Participants were re-categorized by their own assessment of health and the existence or nonexistence of registered illnesses into four categories: subjectively and objectively healthy (SO) with good subjective health and no registered illnesses (*n* = 150), subjectively healthy (S) with good subjective health but with registered illnesses (*n* = 270), objectively healthy (O) with poor subjective health but without registered illnesses (*n* = 160), and unhealthy (UH) with poor subjective health and with registered illnesses (*n* = 679).

### Mortality

Data from all participants who died before January 2017 were obtained from the official Finnish Cause of Death Registry using unique personal identification numbers.

### Institutionalization

Institutionalization was defined as a permanent entry into a nursing home, of which data were gathered from the municipality’s electronic patient record system and coded by month and year of entry.

### Statistical analyses

Differences in categorical baseline characteristics between the groups were tested using the Chi squared test or Fisher’s exact test. Mean ages between two health groups were compared with two-sample t-test and with one-way analysis of variance using Tukey’s method in pairwise comparisons between four health groups.

Hazard ratios (HRs) and their 95% confidence intervals (CI) for mortality and institutionalization were calculated using Cox proportional hazard models. The follow-up periods for mortality analyses were calculated from the baseline measurements to the end of the follow period of 10 and 18 years or to the death of the individual. In institutionalization analyses, the follow-up periods were calculated from the baseline measurements to the end of the follow-period of 10 and 18 years or to the institutionalization of the individual. We used death as a competitive factor in the institutionalization analyses.

Firstly, unadjusted Cox regression analyses were conducted for the association of combined health information with mortality and institutionalization. Secondly, Cox regression analyses were adjusted for age, body mass index (BMI), Mini-Mental State Evaluation (MMSE) scores and education. Also, relative excess risk due to interaction (RERI), attributable proportion due to interaction (AP) and the Synergy Index (S_y_) were calculated for subjective and objective health [22, 23]. *P* values less than 0.05 were considered statistically significant. All statistical analyzes were performed using SAS System for Windows, version 9.4 (SAS Institute Inc., Cary, NC, USA).

## Results

### Baseline characteristics

Baseline characteristics of the participants according to their combined health information are shown in Table 1. There was a largest proportion of young-olds (aged 64–74 years) among the SO group, but it also included participants aged 75 and older. Among the UH, there were participants from all age-groups, however, the participants in the UH were significantly older than those in the SO.

**Table 1:** Baseline characteristics of the participants according to the combined health information (*n* = 1259)

	Combined health information				P value
	Subjectively and objectively healthy (SO) (*n* = 150)	Subjectively healthy^b^ (S) (n = 270)	Objectively healthy^c^ (O) (n = 160)	Unhealthy^d^ (UH) (*n* = 679)	
Age, years^a^	70.5 (5.1) [64–85]	72.1 (5.8) [64–92]	71.5 (6.0) [64–94]	75.1 (7.3) [64–100]	<.001^*^
	n (%)	n (%)	n (%)	n (%)	
Age, years					<.001^*^
64–74	121 (81)	193 (71)	117 (73)	363 (53)	
75–84	25 (17)	69 (26)	37 (23)	229 (34)	
≥ 85	4 (3)	8 (3)	6 (4)	87 (13)	
Gender					0.802
Men	65 (43)	116 (43)	72 (45)	279 (41)	
Women	85 (57)	154 (57)	88 (55)	400 (59)	
MMSE^e^					<.001^*^
≥ 26	133 (89)	217 (80)	122 (76)	431 (63)	
< 26	17 (11)	53 (20)	38 (24)	248 (37)	
Body mass index (*n* = 1255)					<.001^**^
< 20	2 (1)	7 (3)	4 (3)	61 (9)	
20–24.9	42 (28)	68 (25)	45 (28)	189 (28)	
25–29.9	83 (55)	136 (50)	65 (41)	259 (38)	
30–34.9	15 (10)	49 (18)	35 (22)	130 (19)	
≥ 35	8 (5)	10 (4)	11 (7)	36 (5)	
Education					<.001^***^
Basic^f^ or less than basic	116 (77)	222 (82)	142 (89)	633 (93)	
More than basic	34 (23)	48 (18)	18 (11)	46 (7)	
Living situation					0.259
Alone	37 (25)	77 (29)	44 (28)	217 (32)	
With someone	113 (75)	193 (71)	116 (73)	462 (68)	

^a^Values are mean (standard deviation) [range]

^b^Subjectively healthy and objectively unhealthy

^c^Subjectively unhealthy and objectively healthy

^d^Subjectively and objectively unhealthy

^e^Mini-Mental State Evaluation score

^f^Six years of elementary school

^*^SO vs UH, *p* < .001

^**^SO vs O, *p* = 0.025; SO vs UH, p < .001

^***^SO vs O, *p* = 0.007; SO vs UH, p < .001

The MMSE scores in the SO were significantly higher than those in the UH and the BMI profile was significantly better in the SO than in the O and the UH. The SO group had the largest proportion of participants with more than basic education. No significant differences in living situation were found between the groups. No differences in proportions of men and women were found between the groups.

### Follow-up characteristics

After the 10- and 18-year follow-ups, the proportion of deceased participants and the rate of mortality, and the proportion of institutionalized participants and the rate of institutionalization, were higher in the UH than in the SO (Tables 2 and 3).

**Table 2:** Mortality by combined health information during the 10- and 18-year follow-ups (*n* = 1259)

Follow-up period	Combined health information					
		Subjectively and objectively healthy (SO) (n = 150)	Subjectively healthy^a^ (S) (n = 270)	Objectively healthy^b^ (O) (n = 160)	Unhealthy^c^ (UH) (n = 679)	Total
10 years	Deceased n (%)	23 (15)	65 (24)	36 (23)	342 (50)	466 (37)
	Follow-up time, years^d^	9.6 (1.5)	9.0 (2.4)	9.2 (2.0)	7.3 (3.5)	8.2 (3.1)
	Person-years	1441	2419	1471	4949	10,281
	Mortality rate / 1000 person-years	16.0	26.9	24.5	69.1	45.3
18 years	Deceased n (%)	85 (57)	162 (60)	88 (55)	542 (80)	877 (70)
	Follow-up time, years^d^	15.0 (4.2)	13.6 (5.3)	14.3 (5.0)	10.0 (6.2)	11.9 (6.0)
	Person-years	2255	3684	2281	6788	15,008
	Mortality rate / 1000 person-years	37.7	44.0	38.6	79.9	58.4

^a^Subjectively healthy and objectively unhealthy

^b^Subjectively unhealthy and objectively healthy

^c^Subjectively and objectively unhealthy

^d^Values are mean (standard deviation)

**Table 3:** Institutionalization by combined health information during the 10- and 18-year follow-ups (*n* = 1106)

Follow-up period	Combined health information					
		Subjectively and objectively healthy (SO) (*n* = 138)	Subjectively healthy^a^ (S) (*n* = 251)	Objectively healthy^b^ (O) (n = 138)	Unhealthy^c^ (UH) (*n* = 579)	Total
10 years	Institutionalized n (%)	10 (7)	29 (12)	13 (9)	110 (19)	162 (15)
	Follow-up time, years^d^	9.4 (1.8)	8.7 (2.6)	9.0 (2.3)	7.3 (3.4)	8.1 (3.1)
	Person-years	1295	2189	1246	4198	8928
	Institutionalization rate / 1000 person-years	7.7	13.2	10.4	26.2	18.1
18 years	Institutionalized n (%)	28 (20)	76 (30)	37 (27)	187 (32)	328 (30)
	Follow-up time, years^d^	14.3 (4.6)	12.9 (5.4)	13.7 (5.3)	9.7 (6.0)	11.5 (5.9)
	Person-years	1973	3241	1887	5635	12,737
	Institutionalization rate / 1000 person-years	14.2	23.4	19.6	33.2	25.8

^a^Subjectively healthy and objectively unhealthy

^b^Subjectively unhealthy but objectively healthy

^c^Subjectively and objectively unhealthy

^d^Values are mean (standard deviation)

### Cox models for mortality

In unadjusted, and adjusted models, significantly higher mortality rates were found among the UH than that among the SO in both follow-ups (Table 4). The associations were also significant when analyzing only participants followed-up for more than 5 years (Additional file 2). During the 10-year follow-up, also the S had a significantly higher mortality rate than the SO in the unadjusted model, but this did not hold in the adjusted model.

**Table 4:** Association of combined health information and mortality during the 10- and 18-year follow-ups (n = 1259)

Follow-up period	Combined health information				
		Subjectively and objectively healthy (SO)	Subjectively healthy^b^ (S)	Objectively healthy^c^ (O)	Unhealthy^d^ (UH)
10 years	Unadjusted HR (95% CI)	1	1.69 (1.05–2.72)	1.54 (0.91–2.60)	4.40 (2.89–6.72)
	P value		0.030	0.106	<.001
	Adjusted^a^ HR (95%CI) (n = 1255)	1	1.35 (0.83–2.17)	1.29 (0.76–2.19)	2.57 (1.66–3.96)
	P value		0.234	0.371	<.001
18 years	Unadjusted HR (95% CI)	1	1.19 (0.92–1.55)	1.03 (0.76–1.39)	2.32 (1.85–2.92)
	P value		0.194	0.855	<.001
	Adjusted^a^ HR (95%CI) (n = 1255)	1	1.03 (0.79–1.33)	0.92 (0.68–1.24)	1.59 (1.25–2.01)
	P value		0.856	0.588	<.001

HR = Hazard ratio

CI = Confidence interval

^a^Values are adjusted for age, BMI, MMSE scores and education

^b^Subjectively healthy and objectively unhealthy

^c^Subjectively unhealthy and objectively healthy

^d^Subjectively and objectively unhealthy

### Cox models for institutionalization

In unadjusted analyses, the rate of institutionalization was significantly higher in the UH than in the SO during both follow-ups (Table 5). During the 18-year follow-up, the rate of institutionalization was also higher in the S than in the SO. When adjusted for age, BMI, MMSE scores and education, the associations were no longer significant.

**Table 5:** Association of combined health information and institutionalization during the 10- and 18-year follow-ups (n = 1106)

Follow-up period	Combined health information				
		Subjectively and objectively healthy (SO)	Subjectively healthy^b^ (S)	Objectively healthy^c^ (O)	Unhealthy^d^ (UH)
10 years	Unadjusted HR (95% CI)	1	1.65 (0.81–3.36)	1.31 (0.58–2.97)	2.85 (1.50–5.41)
	P value		0.171	0.516	.001
	Adjusted^a^ HR (95%CI) (*n* = 1103)	1	1.21 (0.60–2.45)	1.13 (0.50–2.56)	1.68 (0.88–3.22)
	P value		0.596	0.772	0.116
18 years	Unadjusted HR (95% CI)	1	1.59 (1.04–2.42)	1.37 (0.85–2.22)	1.81 (1.23–2.67)
	P value		0.032	0.194	0.003
	Adjusted^a^ HR (95%CI) (n = 1103)	1	1.35 (0.88–2.07)	1.21 (0.74–1.97)	1.28 (0.86–1.93)
	P value		0.173	0.448	0.227

HR = Hazard ratio

CI = Confidence interval

^a^Values are adjusted for age, BMI, MMSE scores and education

^b^Subjectively healthy and objectively unhealthy

^c^Subjectively unhealthy and objectively healthy

^d^Subjectively and objectively unhealthy

**Fig. 1:**
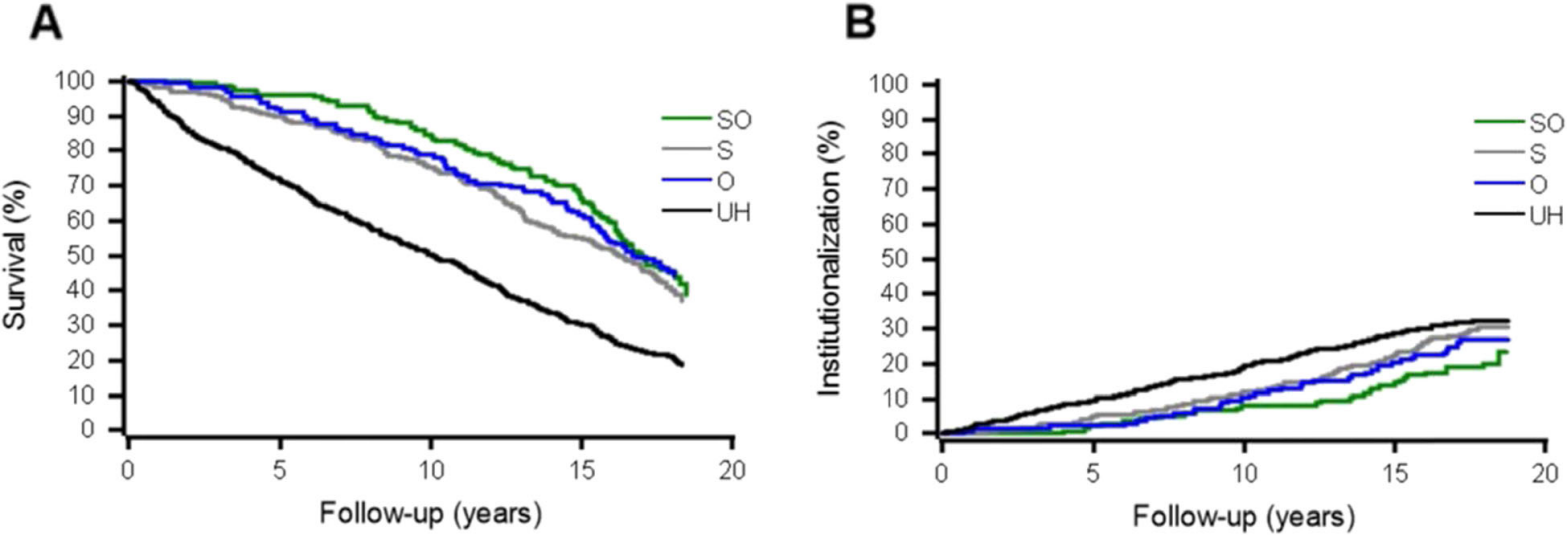
Rates of survival (A) and institutionalization (B) by combined health information (self-reported [subjective] health and registered illnesses [objective health]) during the 18-year follow-up. SO = good subjective and objective health, S = good subjective and poor objective health, O = poor subjective and good objective health and UH = unhealthy, poor subjective and objective health

Figure 1 shows the rates of mortality and institutionalization (with death as a competing risk) by combined health information. Half of the UH were deceased already after 10 years and the mortality continued to increase up till 18 years. The mortality trend can be seen even when including only participants followed-up for more than 5 years (Additional file 3). The higher rate of institutionalization among the UH than that among the other groups can be seen throughout the follow-up period.

### Measures of biological interaction

RERI for mortality was found significant in the unadjusted (RERI 2.17 [95% CI 1.24–3.11] and 1.10 [0.76–1.45] during 10 and 18 years, respectively) and adjusted (0.93 [0.32–1.54] and 0.64 [0.34–0.94] during 10 and 18 years, respectively) analyses during both follow-ups. No statistical significance was found for institutionalization (data not shown).

AP for mortality was found significant in the unadjusted (AP 0.49 [95% CI 0.31–0.68] and 0.48 [0.32–0.63] during 10 and 18 years, respectively) analyses and adjusted (AP 0.36 [0.09–0.63] and 0.40 [0.19–0.62] during 10 and 18 years, respectively) analyses during both follow-ups. No statistical significance was found for institutionalization (data not shown). S_y_ was significant only in the unadjusted analysis on mortality during the 10-year follow-up (S_y_ 2.76 [95% CI 1.28–5.97]).

## Discussion

The rate of mortality was highest in those both subjectively and objectively unhealthy (UH) during both follow-ups. Poor subjective health had an additive effect on poor objective health in predicting mortality.

In earlier studies, the existence of registered illnesses has been shown to predict mortality during a shorter follow-up period [14]. Also, SRH predicted short-term mortality better than long-term [24, 25]. In our study, being objectively and subjectively unhealthy was associated with poor survival even after 18 years. The illnesses were carefully selected and in concordance with the illnesses used in the CCI and the simpler index suggested for primary care [13, 14].

Being subjectively unhealthy but objectively healthy (O), “worried but well”, was not associated with a higher risk of death in the analyses. However, poor subjective health added to the effect of poor objective health in predicting mortality. The additive effect of poor subjective health has also been shown earlier [20]. It is expectable that the individual’s poor subjective health adds to the poor objective health because the mere existence of an illness does not take into account its severity or induced disability. A recent study emphasizes incorporating SRH into the assessment of an older individual’s health as it seems to predict short-term (less than 10 years) mortality almost as well as objective health measured by frailty [24].

After 18 years, there were no longer substantial differences between the proportions of participants still alive in the “super healthy” (SO), the subjectively healthy but objectively unhealthy (S), the “unworried but ill”, and the “worried but well” (O) groups, reflecting perhaps the long follow-up period in which there can be marked changes in an individual’s health. However, the rate of mortality among the UH was still the highest and the AP was still significant after 18 years. This suggests that for mortality, the combination of poor objective and poor subjective health is still notable after 18 years, the result being similar to previous studies with follow-up times of 18 [26], and 27 years [24].

The risk of institutionalization was significantly higher in the UH than in the SO in the unadjusted analyses during both follow-ups, but after adjustments, the differences were no longer statistically significant. The proportion of participants institutionalized was clearly higher in the UH than in the SO in the 10- and 18-year follow-ups, suggesting that the combination of good subjective and objective health (SO) could prevent or delay institutionalization even for a longer time period.

Our findings support the opinion that subjective health can be perceived to include something that objective health can’t [2], or it could be also argued that the individual’s attitude, positive or negative, towards their own health could have an impact on future adverse effects, such as mortality and institutionalization. Thus the psychosocial aspect of an older person’s wellbeing is important to acknowledge as well.

In an earlier study, simple SRH was found to be a better predictor of institutionalization than registered illnesses [18]. The same study found also that the existence of registered illnesses affects the SRH, but through subjective health complaints, not directly. Another study found that being subjectively unhealthy although objectively healthy (“worried but well”) had a higher risk of institutionalization [20]. In our study, the rates of institutionalization were not significantly higher in the other three groups than in the “super healthy” (SO) in the adjusted analyses.

When analyzing the risk of institutionalization, the complexity of factors leading to institutionalization has to be considered. The existence of an illness does not need to affect the individual’s ability to continue living at home, when at the same time it might clearly increase the individual’s risk of death. Higher age, living alone, low BMI, multiple falls, depression, and cognitive and functional impairment have been shown to predict institutionalization in the elderly [27–29]. Also other factors, such as use of formal and informal care, influence institutionalization [29–31], and these were not considered here.

The strengths of this study are the large sample size of a community-dwelling population, high participation rate and the long follow-up-period. The dates of institutionalization were gathered from the electronic patient record system and are therefore more exact compared to previous studies [17, 27, 32, 33]. The more comprehensive self-evaluation of health used in this study including satisfaction in life and self-reported ability to walk 400 m, in addition to simple SRH, was in line with the World Health Organization’s definition of health [34]. The participants in the UH group were older and had lower BMI and MMSE scores, which are risk factors for mortality and institutionalization [28, 29, 35, 36]. They were also less educated. We therefore adjusted the analyses for age, BMI, MMSE scores and education, and there still were significant differences in mortality between the “super healthy” (SO) and the unhealthy (UH) during both follow-ups. After adjustments, the differences in institutionalization between these groups were no longer significant, reflecting perhaps the multifactoriality of institutionalization.

Analyzing the risk of death and institutionalization on the basis of only baseline information is a limit to this study. This might affect the analyses on institutionalization even more than on mortality, as institutionalization is more multifactorial.

## Conclusions

In this study, we found the categorization of subjective and objective health into four health groups to be good at predicting the risk of mortality during 10- and 18-year follow-ups. We also found that subjective health had an additive effect on objective health in regarding the risk of mortality. We plan to investigate further which illnesses and if multimorbidity defined using different cut-off points increase the risk of institutionalization in this population, when accounting for also the illnesses acquired during the follow-up period.

## Supplementary Information

**Additional file 1.** Registered illnesses used in the stud.

**Additional file 2.** Associations of combined health information and mortality in participants followed-up for more than 5 years (*n* = 1019).

**Additional file 3.** Rates of survival by combined health information (self-reported [subjective] health and registered illnesses [objective health]) for participants followed-up for more than 5 years, during the 18-year follow-up. SO = good subjective and objective health, S = good subjective and poor objective health, O = poor subjective and good objective health and UH = unhealthy, poor subjective and objective health.

## Data Availability

The datasets used and/or analyzed during the current study are available from the corresponding author on reasonable request.

## Abbreviations

BMI: Body mass index
CCI: Charlson Comorbidity Index
CI: Confidence intervals
HR: Hazard ratio
MMSE: Mini-Mental State Examination
O: Objectively healthy
PRH: Physician rated health
S: Subjectively healthy
SD: Standard deviation
SO: Subjectively and objectively healthy
SRH: Self-rated health
UH: Unhealthy
