# Supplementary material for: Subjective and objective health predicting mortality and institutionalization: an 18-year population-based follow-up study among community-dwelling Finnish older adults
Source: BMC Geriatr. 2021 Jun 10;21:358. doi: 10.1186/s12877-021-02311-w (PMC8193868; doi:10.1186/s12877-021-02311-w)
Supplement: Supplementary file 1 — Additional file 1. Registered illnesses used in the stud. [file 12877_2021_2311_MOESM1_ESM.docx]

| ICD-10 | Registered illness |
| --- | --- |
| C0‒C97  (except C44.01, C44.11, C44.21, C44.31, C44.41, C44.51, C44.61, C44.71, C44.81, C44.91) | Malignant neoplasms (except basal cell carcinomas) |
| E10‒E14 | Diabetes mellitus |
| F00‒F03 | Dementia |
| F10 | Alcohol induced disorders |
| F19 | Drug use induced disorders |
| F30‒F39 | Mood disorders |
| G10‒G26 | Systemic atrophies, extrapyramidal and movement disorders |
| G30 | Alzheimer’s disease |
| G35 | Multiple Sclerosis |
| G45 | Transient cerebral ischemic attack |
| G62 | Other polyneuropathies |
| I11 | Hypertensive heart disease |
| I20‒I25 | Ischemic heart disease |
| I34‒I37 | Valve disorders |
| I48 | Atrial fibrillation |
| I60‒I69 | Cerebrovascular disorders |
| I70 | Atherosclerosis |
| I73.9 | Peripheral vascular disease, unspecified |
| J40‒J47 | Chronic lower respiratory diseases |
| M05‒M06 | Rheumatoid arthritis |
| M45 | Ankylosing spondylitis |
| N17‒N19 | Renal failure |

Additional file 1. Registered illnesses used in the study.
