# Supplementary material for: Subjective and objective health predicting mortality and institutionalization: an 18-year population-based follow-up study among community-dwelling Finnish older adults
Source: BMC Geriatr. 2021 Jun 10;21:358. doi: 10.1186/s12877-021-02311-w (PMC8193868; doi:10.1186/s12877-021-02311-w)
Supplement: Supplementary file 2 — Additional file 2. Associations of combined health information and mortality in participants followed-up for more than 5 years (n = 1019). [file 12877_2021_2311_MOESM2_ESM.docx]

|  |  | Combined health information | | | |
| --- | --- | --- | --- | --- | --- |
| Follow-up period |  | Subjectively and objectively healthy (SO) | Subjectively healthy^b^ (S) | Objectively healthy^c^ (O) | Unhealthy^d^ (UH) |
| 10 years | Unadjusted HR  (95% CI) | 1 | 1.39  (0.78‒2.46) | 1.37  (0.73‒2.57) | 2.95  (1.79‒4.87) |
|  | P value |  | 0.262 | 0.320 | <.001 |
|  | Adjusted^a^ HR  (95%CI) (n=1017) | 1 | 1.35  (0.83‒2.17) | 1.29  (0.76‒2.19) | 2.57  (1.66‒3.96) |
|  | P value |  | 0.234 | 0.371 | <.001 |
| 18 years | Unadjusted HR  (95% CI) | 1 | 1.09  (0.83 ‒1.44) | 0.96  (0.70‒1.31) | 1.81  (1.41‒2.31) |
|  | P value |  | 0.532 | 0.778 | <.001 |
|  | Adjusted^a^ HR  (95%CI) (n=1017) | 1 | 0.96  (0.73‒1.27) | 0.89  (0.65‒1.22) | 1.37  (1.06‒1.76) |
|  | P value |  | 0.779 | 0.460 | 0.015 |

Additional file 2. Association of combined health information and mortality in participants followed-up for more than 5 years (n=1019)

HR = Hazard ratio

CI = Confidence interval

^a^Values are adjusted for age, BMI, MMSE scores and education

^b^Subjectively healthy and objectively unhealthy

^c^Subjectively unhealthy and objectively healthy

^d^Subjectively and objectively unhealthy
