# Supplementary material for: Subjective and objective health predicting mortality and institutionalization: an 18-year population-based follow-up study among community-dwelling Finnish older adults
Source: BMC Geriatr. 2021 Jun 10;21:358. doi: 10.1186/s12877-021-02311-w (PMC8193868; doi:10.1186/s12877-021-02311-w)
Supplement: Supplementary file 3 — Additional file 3. Rates of survival by combined health information (self-reported [subjective] health and registered illnesses [objective health]) for participants followed-up for more than 5 years, during the 18-year follow-up. SO = good subjective and objective health, S = good subjective and poor objective health, O = poor subjective and good objective health and UH = unhealthy, poor subjective and objective health. [file 12877_2021_2311_MOESM3_ESM.docx]

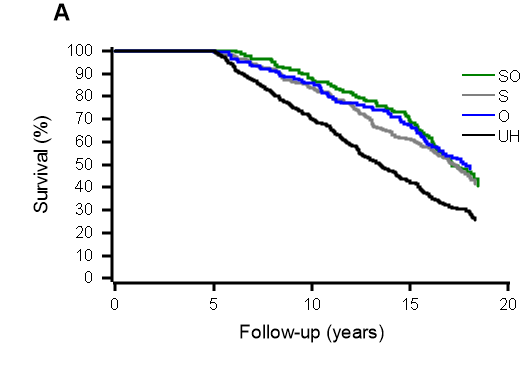


Additional file 3. Rates of survival by combined health information (self-reported [subjective] health and registered illnesses [objective health]) for participants followed-up for more than 5 years, during the 18-year follow-up. SO = good subjective and objective health, S = good subjective and poor objective health, O = poor subjective and good objective health and UH = unhealthy, poor subjective and objective health.
